# Genome-Wide Analysis of DNA Methylation Profiles on Jining Grey Goat Uterus Associated with Prolificacy Using Whole-Genome Bisulfite Sequencing

**DOI:** 10.3390/ani16182935

**Published:** 2026-09-18

**Authors:** Cunming Yang, Junmin He, Jingyi Mao, Xue Li, Guifen Liu, Guoping Zhang, Chen Wei, Yifan Ren, Kechuan Tian, Xixia Huang

**Affiliations:** 1 Shandong Key Laboratory of Livestock and Poultry Breeding, Institute of Animal Husbandry and Veterinary Medicine, Shandong Academy of Agricultural Sciences, Jinan 250100, China; yangcunming0405@163.com (C.Y.);; 2 College of Animal Science, Xinjiang Agricultural University, Urumqi 830052, China; 3 College of Life Sciences, Xinjiang Normal University, Urumqi 830017, China

**Keywords:** Jining Grey Goat, litter size, uterus, whole-genome bisulfite sequencing, differentially methylated genes

## Abstract

Litter size is a vital economic trait in goat production, and previous studies on its regulatory mechanisms mainly focus on ovarian tissues. The genome-wide DNA methylation landscape of the goat uterus and its association with prolificacy remain poorly understood. In this study, we performed whole-genome bisulfite sequencing (WGBS) on uterine tissues from high-prolificacy (HP) and low-prolificacy (LP) Jining Grey Goats to explore epigenetic regulatory mechanisms underlying litter size. A total of 880 differentially methylated regions (DMRs) and 274 differentially methylated genes (DMGs) were identified. Combined with transcriptome data, 21 genes were found to exhibit both differential methylation and differential expression. Three candidate genes (*CFAP43*, *PLPPR1* and *TRPC3*) and their corresponding DMRs were further verified via bisulfite sequencing PCR (BSP). Integrated analysis highlighted *CFAP43*, *TTC21A*, and *CROCC* as key candidate genes functionally associated with ciliary structure and maintenance, whose methylation and expression patterns correlate with the litter size phenotype. This study establishes the first genome-wide DNA methylation map of the Jining Grey Goat uterus. We demonstrate that DNA methylation modulates the expression of *CFAP43*, *TTC21A* and *CROCC*, and further regulates uterine ciliary function to affect goat prolificacy. Our findings provide novel epigenetic insights into goat reproductive traits and supply candidate molecular markers for goat breeding.

## Simple Summary

Goat breeding is an important industry worldwide, and raising the number of kids per birth can greatly raise farmers’ income. Improving goat litter size through conventional breeding alone remains challenging, as this complex trait is governed by intricate interactions among genetic, endocrine, and environmental factors. The uterus is the organ where embryos implant and develop, yet few studies have explored how DNA methylation in the uterus influences litter size. We chose a local Chinese goat breed famous for bearing many kids, and compared the genetic modification patterns in the womb tissue of goats that produce many lambs and those that produce only one kid each time. We found three key genes that control the normal structure and function of tiny hair-like structures inside the womb. Changes to these genes through genetic modification can affect how well the womb supports embryo growth, and finally change the number of newborn lambs. The findings explain why some goats have more babies, and provide practical genetic markers to help breeders select goats that produce larger litters, bringing stable economic gains to goat farmers.

## 1. Introduction

The goat farming industry constitutes a significant component of the global agricultural economy. Herd sizes are continually increasing, underscoring its importance for food security and economic development [1,2,3]. China, which has the world’s largest goat inventory, is witnessing consistent expansion within its industry chain. Litter size is a central determinant of reproductive performance in goats, and its improvement directly enhances farming profitability [4]. However, litter size is a quintessential low-heritability trait. Its phenotypic variation is controlled by the polygenic synergistic effects of numerous genes, combined with environmental influences, and encompasses a complex sequence of biological events including hormone secretion, folliculogenesis, embryo implantation, and fetal development [5,6,7,8]. These reproductive biological processes are precisely regulated by epigenetic modifications, among which DNA methylation, as the most well-characterized epigenetic mark, plays a pivotal regulatory role in mammalian reproductive physiology. Nevertheless, systematic research on DNA methylation-mediated regulatory mechanisms underlying goat litter size remains largely insufficient to date.

DNA methylation is established as a crucial epigenetic modification that plays a fundamental role in regulating reproductive processes [9]. Research indicates that dynamic, genome-wide alterations in DNA methylation patterns occur during germ cell specification, gametogenesis, and pre-implantation development, highlighting its importance as a key regulatory mechanism in reproduction [10,11]. While initial progress has been made in understanding the role of DNA methylation in goat prolificacy, research remains limited and has primarily focused on ovarian tissue. For example, studies involving Guanzhong dairy goats and Jining Grey Goats have analyzed ovarian DNA methylation profiles, suggesting genes such as *NR5A2*, *STAR*, *FGF2*, *BMP5*, and *CFAP43* as potential regulators of estrus and litter size [12,13,14]. In the caprine uterus, evidence suggests that *METTL3* modulates endometrial epithelial cell proliferation via regulation of connective tissue growth factor [15]. Additional studies targeting specific genes have reported associations between litter size and promoter region polymorphisms/methylation status of the caprine *KISS1* gene [16], as well as significant correlations between genetic variants in the *DNMT3B* gene and litter size in Shanbei White Cashmere goats [17]. Collectively, current studies on DNA methylation in caprine uterine tissues are mostly focused on single-gene level targeted exploration, and no study has systematically mapped the genome-wide DNA methylation landscape of goat uterine tissue. As a core reproductive organ for embryo implantation, pregnancy establishment and full-term maintenance, the functional homeostasis of the uterus is a key factor determining pregnancy outcomes and ultimately affecting the litter size phenotype. However, to date, the genome-wide DNA methylation profile of the goat uterus and its epigenetic regulatory mechanism underlying the divergence of litter size traits between high- and low-prolificacy goats remain largely uncharacterized.

Given this critical research gap, and that the uterus is a vital reproductive organ alongside the ovaries [18], elucidating the molecular mechanisms through which DNA methylation influences goat reproductive efficiency is of paramount importance. The Jining Grey Goat, distinguished by its high fecundity and year-round prolificacy, serves as a unique and valuable model for investigating the genetic and epigenetic basis of mammalian reproductive traits [8,12,14]. We hypothesized that differential DNA methylation in uterine tissue contributes to the divergence in litter size between high- and low-prolificacy Jining Grey Goats by regulating the expression of genes involved in endometrial function, ciliary activity, and embryo implantation. This study was designed to characterize and compare the genome-wide DNA methylation landscapes of uterine tissues from high- and low-fertility Jining Grey Goats, and to explore how differential methylation might regulate key gene expression to ultimately influence litter size. We performed WGBS on estrus-phase uterine tissues from Jining Grey Goats with divergent litter sizes to generate comprehensive methylation profiles. Integrating these data with previously acquired transcriptomic (RNA-seq) data from our group allowed for direct correlation of methylation changes with gene expression alterations. Our objectives are to identify critical DMRs and DMGs associated with litter size, to preliminarily investigate their potential regulatory functions, to provide novel epigenetic insights into the mechanisms underlying caprine prolificacy, and to identify promising candidate targets for application in molecular breeding strategies.

## 2. Materials and Methods

### 2.1. Selection of Animals and Tissue Collection

Experimental animals were selected from the National Jining Grey Goat Breeding Farm in Jiaxiang County, Jining City, Shandong Province, China. A total of eight healthy, age- and weight-matched Jining Grey Goats were raised under standardized management conditions and assigned to either a high-prolificacy (HP) group (*n* = 4; ≥3 kids per litter for three consecutive pregnancies) or a low-prolificacy (LP) group (*n* = 4; exactly 1 kid per litter for three consecutive pregnancies). Estrus was synchronized in all ewes using an intravaginal CIDR (Zoetis Inc, Parsippany, NJ, USA; Cat. No. 05414736051093) device. The CIDR was removed after 14 days, followed by an intramuscular injection of 300 IU of pregnant mare serum gonadotropin (PMSG, NSNF; Cat. No. 220118). Estrus status was confirmed by vaginal examination as previously described [14]. To ensure standardized sampling across all synchronized individuals, uterine tissues were collected within 24 h after the onset of estrus. Tissues were immediately frozen in liquid nitrogen, and stored at −80 °C until total DNA and RNA extraction. All experimental procedures were reviewed and approved by the Animal Ethics Committee of the Institute of Animal Science and Veterinary Medicine, Shandong Academy of Agricultural Sciences (Approval No.: IASVM-2022-003).

### 2.2. DNA Extraction

Genomic DNA was extracted from uterine tissues using the TIANamp Genomic DNA Kit (Tiangen Biotech, Beijing, China; Cat. No. DP304), with all operations performed strictly following the manufacturer’s standard protocol. The integrity of genomic DNA was verified via 2% agarose gel electrophoresis, and the concentration and purity (A260/A280 and A260/A230 ratios) were quantified using a NanoDrop™ One spectrophotometer (Thermo Scientific, Waltham, MA, USA). All qualified DNA samples were stored at −80 °C until subsequent library construction and sequencing analysis.

### 2.3. WGBS Library Preparation, Sequencing, and Methylation Calling

Qualified genomic DNA was fragmented by sonication into 200–300 bp fragments, followed by end repair, adenylation, and adapter ligation. Bisulfite conversion was performed using the EZ DNA Methylation-Gold™ Kit (Zymo Research Corporation, Orange, CA, USA; Cat. #D5005). The resulting libraries were purified, size-selected by gel extraction, and amplified by PCR to generate final sequencing libraries. Libraries were sequenced on the MGISEQ-2000 platform (MGI Tech Co., Ltd., Shenzhen, China). Raw sequencing reads were processed with SOAPnuke (v1.5.6) to remove adapter-containing and low-quality reads (defined as those with >1% N bases or >40% of bases having a Phred quality score ≤20). Clean reads were obtained, and Q30 scores and GC content were calculated.

Clean reads were aligned to the goat reference genome (ARS1, GCF_001704415.1) using Bismark (v0.23.0) [19]. After duplicate removal (duplicate rates ranged from 2.04% to 2.99% across samples), cytosine methylation calls and methylation levels were extracted using the bismark_methylation_extractor and coverage2cytosine tools [20,21]. CpG sites with a minimum coverage of 5× were retained for subsequent analysis. The average sequencing depth was approximately 30× per sample, with an average CpG coverage of approximately 10×. Differentially methylated regions (DMRs) were identified using the R package DMRcaller (v1.30.0) [22] with the following thresholds: |Δβ| ≥ 0.2 for CG, ≥0.1 for CHG, and ≥0.05 for CHH contexts. Resulting *p*-values were corrected for multiple testing using the Benjamini–Hochberg false discovery rate (FDR) method, and regions with adjusted *p* ≤ 0.05 were retained as DMRs. DMRs were annotated using BEDTools (v2.30.0) [23], with regions overlapping genes or functional elements by at least 1 bp being annotated as associated DMRs.

### 2.4. Bisulfite Sequencing PCR (BSP)

BSP was employed to validate the methylation levels of key DMRs. Genomic DNA was subjected to bisulfite conversion using the EZ DNA Methylation-Gold™ Kit (Zymo Research, USA; Cat. #D5005). The converted DNA was eluted and diluted to a final concentration of 200 ng/μL. BSP primers targeting key DMRs were designed using MethPrimer 2.0 software and synthesized by Sangon Biotech (Shanghai, China). PCR amplification was performed using the bisulfite-converted DNA as template. The resulting PCR products were evaluated by agarose gel electrophoresis and purified using a PCR Purification Kit (Bioer Technology, Hangzhou, China; Cat. #BSC02M1A). The purified products were subsequently cloned into the pMD19-T vector (Takara, Osaka, Japan; Cat. #6013) and transformed into DH5α competent cells (AngYuBio, Shanghai, China; Cat. #G6016). For each sample, three positive clones were randomly selected for Sanger sequencing by Sangon Biotech. The sequencing results were aligned to the reference genome using BiQ Analyzer HT v3.0, and the methylation percentage at each CpG site was calculated. The primer sequences used for BSP are listed in Table 1.

### 2.5. Bioinformatics Analysis

Gene Ontology (GO) and KEGG pathway enrichment analyses for DMR-associated genes (DMGs) were performed using DAVID 6.8 [24], with a significance threshold of *p* < 0.05. Protein–protein interaction (PPI) networks were constructed based on the STRING database (v12.0), retaining only interactions with a confidence score >0.7. The differential PPI network was visualized using the igraph R package alongside ggraph, dplyr, and RColorBrewer for enhanced graphical representation. For sample-to-sample correlation analysis, the chromosome-level CG methylation rates of all eight samples were extracted and merged into a sample-by-chromosome matrix. Pearson correlation coefficients were calculated between every pair of samples using the cor function in R (v4.2.1), and the resulting correlation matrix was visualized as a heatmap using the pheatmap R package (v1.0.12).

## 3. Results

### 3.1. Construction of Genome-Wide DNA Methylation Profiles in Uterine Tissues of Jining Grey Goats

Genomic DNA was extracted from uterine tissues of Jining Grey Goats. The concentration and purity of all samples met the established standards, ensuring high quality for subsequent analysis. WGBS was then performed to systematically construct genome-wide DNA methylation maps of the Jining Grey Goat uterus.

After stringent quality control, approximately 81 Gb of clean reads were obtained per sample on average, corresponding to an average sequencing depth of approximately 30× and an average CpG coverage of approximately 10×. Q20 scores ranging from 96.00% to 97.55% and Q30 scores ranging from 87.97% to 93.34%. The unique mapping rate of reads to the goat reference genome ranged from 72.00% to 83.13% across all samples. The bisulfite conversion efficiency exceeded 99% for all libraries. These metrics confirmed the high reliability of the sequencing data, supporting their use in subsequent analyses.

To assess the reproducibility and reliability of the WGBS data, Pearson correlation analysis was performed based on the chromosome-level CG methylation rates across all eight biological samples. The results showed high correlation coefficients among all samples (Pearson r = 0.994–0.999), with average r values of 0.998 within the HP group, 0.997 within the LP group, and 0.998 between the HP and LP groups (Figure S1). These findings demonstrated excellent biological reproducibility and high reliability of the WGBS dataset, supporting its suitability for subsequent differential methylation analysis.

**Figure 1:**
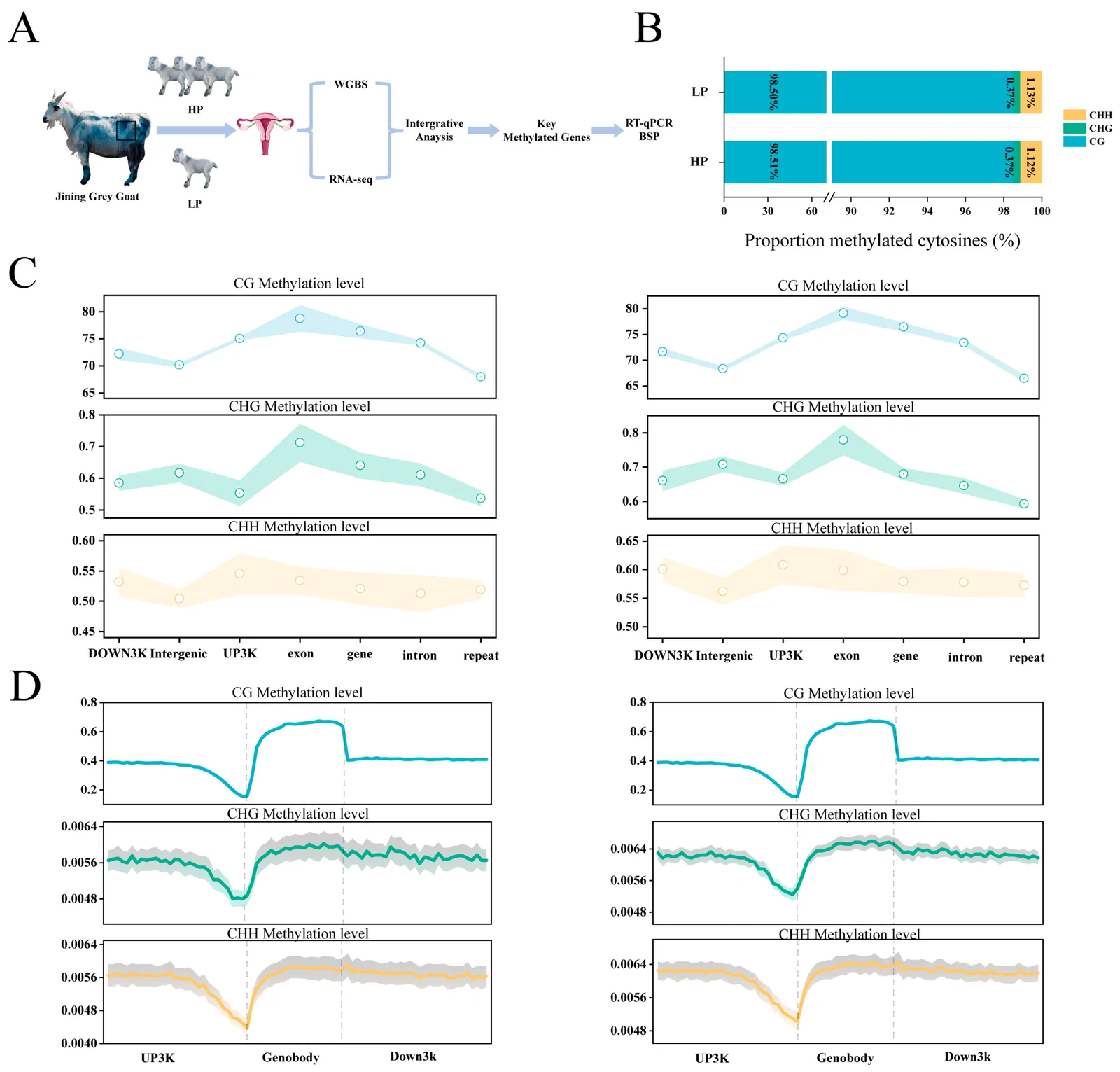
Characterization of DNA methylation in uterine tissues of Jining Grey Goat. (**A**) Schematic diagram of experimental design. (**B**) Stacked bar plot showing the average proportion of mCG, mCHG and mCHH in uterine tissues. (**C**) Methylation levels across distinct genomic features. The *x*-axis represents genomic elements, and the *y*-axis indicates methylation level. Blue, green and yellow lines correspond to CG, CHG and CHH methylation, respectively. (**D**) Methylation distribution across regions spanning 3 kb upstream to 3 kb downstream of transcription sites. The left and right panels show data from the HP and LP groups, respectively. HP, high-prolificacy group; LP, low-prolificacy group.

Methylation analysis revealed that approximately 2.83% to 4.08% of all cytosine (C) bases were methylated in the goat uterine genome (Figure 1B). Methylation in the CG context (mCG) was the most predominant, accounting for over 98% of total methylation. In contrast, methylation levels in the CHG and CHH contexts (where H represents A, C, or T) were substantially lower, ranging from 0.36% to 0.38% and 1.05% to 1.17%, respectively. No significant differences in these patterns were observed between the high- and low-fertility groups (all *p* > 0.05), indicating that mCG is the principal form of DNA methylation in uterine tissue (Table S1).

In the mCG context, methylation levels varied across distinct genomic features (Figure 1C). Exonic regions exhibited the highest methylation levels (approximately 0.78), whereas repetitive regions showed the lowest (0.66–0.68), with no significant differences observed between the HP and LP groups (all *p* > 0.05). Analysis of methylation patterns across gene bodies revealed a gradual decrease from approximately 0.37 at 3 kb upstream of the transcription start site (TSS) to a minimum of about 0.15 near the TSS. Upon entering the gene body region, methylation levels increased significantly, exceeding 0.65 and indicating a hypermethylated state. Downstream of the transcription termination site (TTS), methylation levels decreased again to approximately 0.4 at 3 kb downstream (Figure 1D). Notably, negligible methylation signals were detected in the CHG and CHH contexts across all analyzed samples.

### 3.2. Identification of DMRs and DMGs in Uterine Tissues of Jining Grey Goats with Divergent Litter Size

**Figure 2:**
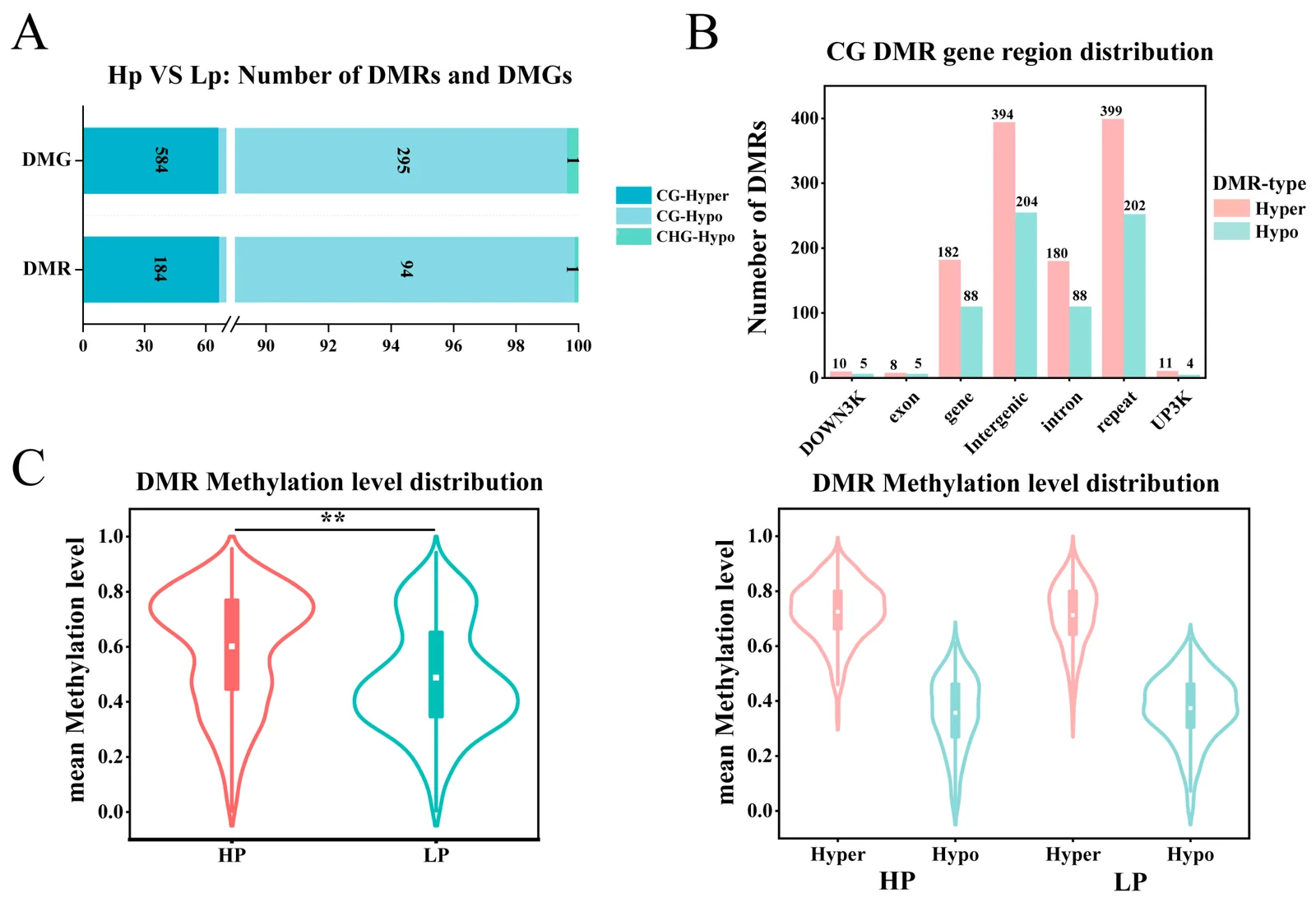
Identification of differentially methylated regions (DMRs) and differentially methylated genes (DMGs) in goat uterine tissue. (**A**) Stacked bar plots showing the numbers of DMRs and DMGs in CG and CHH contexts. (**B**) Genomic distribution of CG-DMRs. The x-axis indicates different genomic regions, and the y-axis represents the number of DMRs. (**C**) Violin plots displaying the mean methylation levels of CG-DMRs in HP and LP groups. **, *p* < 0.01.

Using the high-prolificacy (HP) group as the experimental group and the low-prolificacy (LP) group as the control, a total of 880 differentially methylated regions (DMRs) were identified. The vast majority (>99%) of these DMRs occurred in the CG sequence context (CG-DMRs: 584 hypermethylated, 295 hypomethylated). Only one hypomethylated DMR was identified in the CHG context. These DMRs were annotated to 274 genes (CG-associated: 184 hypermethylated, 94 hypomethylated; CHG-associated: 1 hypomethylated), confirming the absolute predominance of the CG context in both DMRs and differentially methylated genes (DMGs) (Figure 2A).

Given the dominance of CG-context methylation, subsequent analyses focused exclusively on this context. Annotation of CG-DMRs to different genomic functional regions revealed that DMRs were most densely distributed in repetitive sequences, with only a small proportion located in regions 3 kb downstream of genes (DOWN3K), exons, or 3 kb upstream of genes (UP3K) (Figure 2B). The distribution of methylation levels within CG-DMRs showed that the average methylation level in the HP group was predominantly between 0.6 and 0.85, whereas in the LP group it concentrated in the lower range of 0.2–0.6. Methylation was significantly higher in the HP group than in the LP group (*p* < 0.01). Accordingly, the HP group exhibited a higher proportion of hypermethylated DMRs, while the LP group showed a greater proportion of hypomethylated DMRs (Figure 2C).

### 3.3. Screening and Analysis of Litter Size-Associated Differentially Methylated Genes in Uterine Tissue

To systematically elucidate the biological functions of the identified DMGs in uterine tissue and their potential association with prolificacy, we performed Gene Ontology (GO) annotation, KEGG pathway enrichment analysis, and protein–protein interaction (PPI) network analysis on the 273 DMGs.

**Figure 3:**
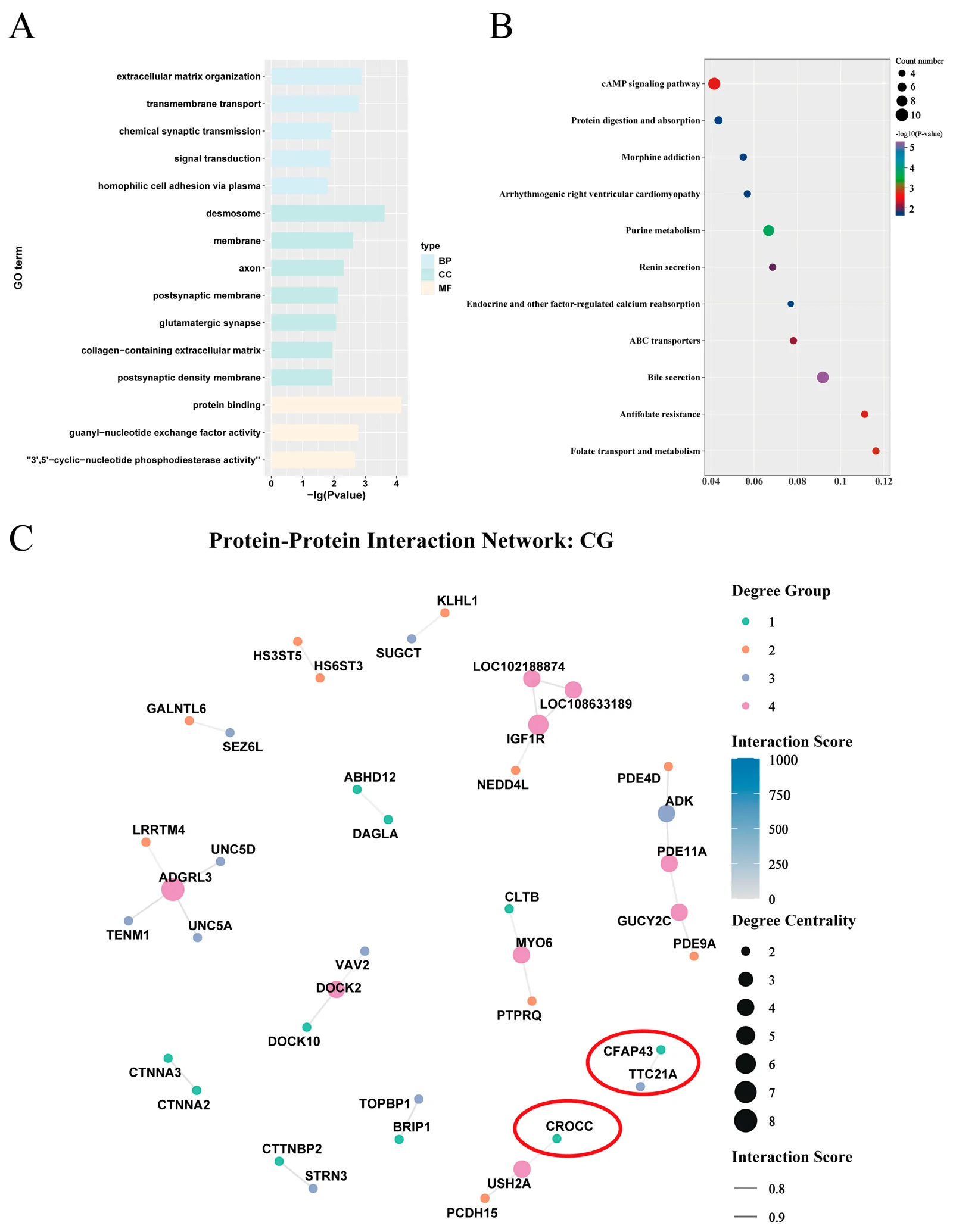
Functional enrichment and protein–protein interaction (PPI) analysis of DMGs. (**A**) Top 15 significantly enriched GO terms covering biological process (BP), cellular component (CC) and molecular function (MF). (**B**) KEGG pathway enrichment bubble plot. Dot size represents the number of DMGs, and color indicates enrichment significance. (**C**) PPI network of DMGs. Edge thickness and color stand for interaction confidence; node size is proportional to degree centrality. Red nodes represent the overlapping genes of DMGs and DEGs, including *CFAP43*, *TTC21A* and *CROCC*.

GO enrichment analysis revealed significant enrichment across all three categories: Biological Process (BP), Cellular Component (CC), and Molecular Function (MF) (Figure 3A). Within BP, the DMGs were primarily involved in processes closely related to cellular communication and microenvironment regulation, including extracellular matrix organization, transmembrane transport, chemical synaptic transmission, and cell signal transduction. For CC, the enriched terms were highly concentrated in the plasma membrane and its specialized structures, such as desmosomes, membranes, and glutamatergic synapses. In the MF category, the DMGs were significantly enriched for protein binding, guanine nucleotide exchange factor (GEF) activity, and 3′,5′-cyclic-nucleotide phosphodiesterase activity, suggesting these genes play important roles in mediating protein interactions and regulating key second messenger metabolism.

KEGG pathway analysis further uncovered specific physiological processes and signaling networks involving the DMGs (Figure 3B). Significantly enriched pathways included bile secretion, folate absorption and metabolism, antifolate resistance, the cAMP signaling pathway, and ABC transporter function. These pathways are intimately linked to reproduction-related processes such as nutrient transport, signal transduction, and cellular energy metabolism.

The PPI network, constructed using the STRING database, indicated extensive functional collaboration among the DMGs (Figure 3C). Notably, three genes—*CFAP43*, *TTC21A*, and *CROCC*—were identified as both differentially methylated and differentially expressed genes (DEGs). Among them, *CFAP43* and *TTC21A* interacted with each other and were co-enriched for the “protein binding” function. *CROCC* formed another interaction module with *USH2A* and *PCDH15*. This network was significantly enriched for components related to “membrane” and “cell membrane”, implying a potential role in maintaining cellular structure and facilitating membrane-related signaling.

### 3.4. Integrated WGBS and RNA-Seq Analysis and Validation

**Figure 4:**
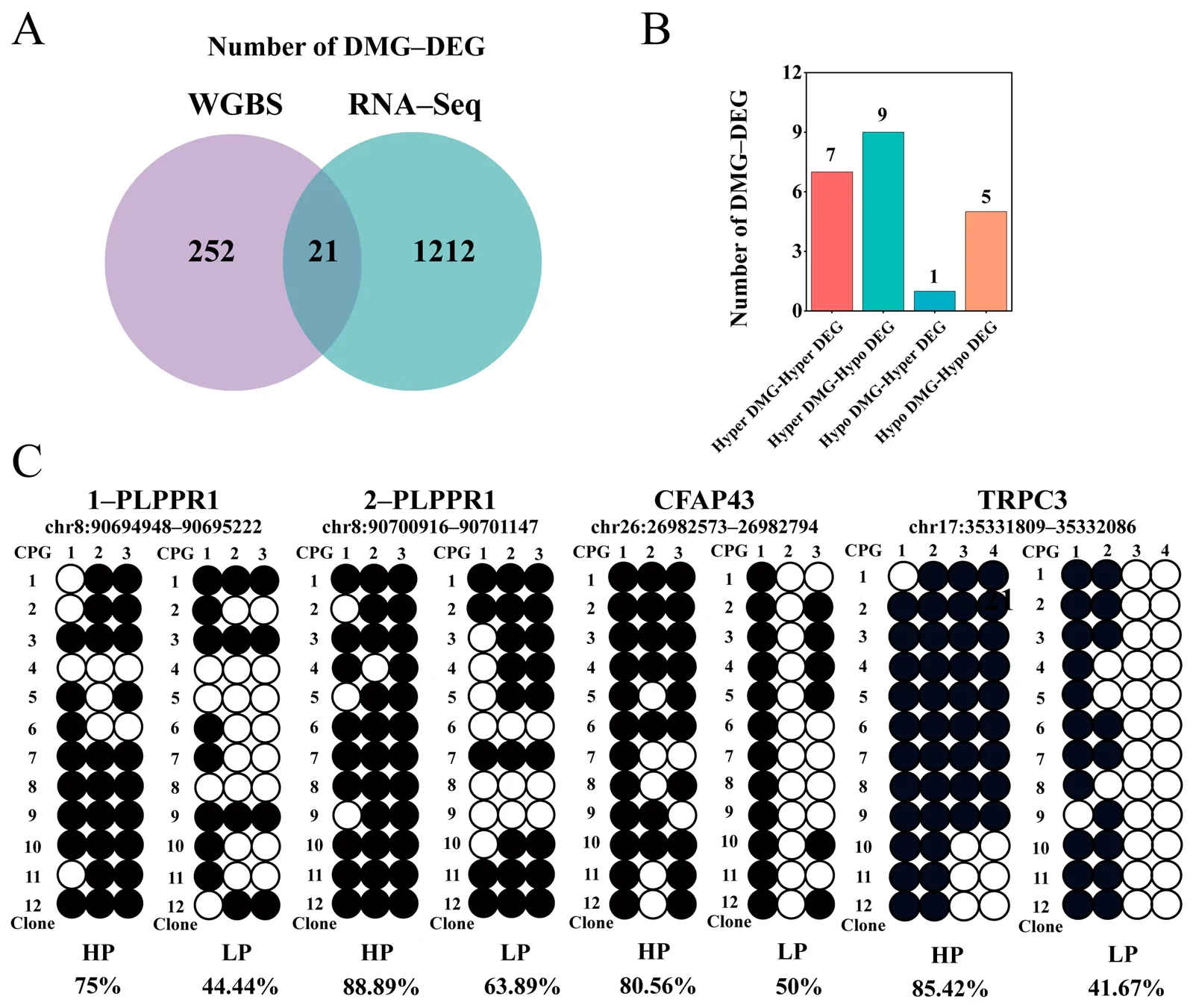
Integrative analysis of WGBS and RNA-seq data combined with BSP validation. (**A**) Venn diagram showing the overlap between DMGs (purple) and DEGs (cyan). (**B**) Classification of DMG-DEG pairs based on methylation and expression trends. (**C**) Methylation status of four representative DMRs validated by BSP. Each row indicates an individual clone. Filled and open circles represent methylated and unmethylated CpG sites, respectively.

To systematically investigate the potential regulatory role of DNA methylation on gene expression, we integrated data from WGBS and RNA sequencing (RNA-seq). A total of 273 DMGs were identified in this study. From the uterine transcriptome data, 1233 DEGs were selected based on the thresholds of |log_2_FC| ≥ 1 and Q ≤ 0.05 (Figure 4A). Comparative analysis revealed 21 genes that exhibited both differential methylation and differential expression (DMG-DEGs) (Table S5). Among these, the methylation changes of 12 genes were concordant with the direction of their expression changes, while 10 genes showed an inverse correlation (Figure 4A,B), suggesting that their expression may be regulated by DNA methylation (Table S2).

To validate the reliability of these multi-omics findings, we randomly selected three inversely correlated genes—*PLPPR1*, *CFAP43*, and *TRPC3*—for experimental verification, based on their significant differential methylation and expression (*p* < 0.05), their potential roles in reproductive-related processes, and the feasibility of BSP primer design. BSP primers were designed targeting the corresponding DMRs of these genes; due to constraints in primer specificity and CpG density, the optimal primer pairs yielded amplicons that largely overlapped with and essentially covered the target DMRs. The validated regions included two DMRs for *PLPPR1* (chr8:90694948-90695222 and chr8:90700916–90701147), one for *CFAP43* (chr26:26982573-26982794; BSP amplicon: chr26:26982573–26982794), and one for TRPC3 (chr17:35331809-35332086). We assessed their methylation status using BSP.

BSP validation showed that the methylation levels of all four DMRs were significantly elevated in the HP group compared with the LP group (Figure 4C), which was fully consistent with the differential methylation trends detected by WGBS (Table S3). These results confirmed the high accuracy of our genome-wide DNA methylation profiling and DMR screening workflow, providing a robust experimental basis for further functional analysis of these differential methylation regions in regulating goat prolificacy.

### 3.5. Screening and Characterization of Key Methylated Genes

Through integrated multi-omics and PPI network analysis, we identified three key nodal DMG-DEGs—*CFAP43*, *TTC21A*, and *CROCC*—and further investigated their potential functions by combining GO and KEGG enrichment results. *CFAP43* and *TTC21A* were primarily enriched for the “protein binding” function, while *CROCC* was significantly associated with “cell membrane”-related components (Table S4).

These three genes were collectively associated with four DMRs located in intronic regions. Their genomic coordinates are *CFAP43* (chr26:26982601-26982800; chr26:27046601–27046800), TTC21A (chr22:12323601–12323800), and *CROCC* (chr2:558401–558600). Methylation-expression correlation analysis revealed that the methylation levels of *CFAP43* and *CROCC* were negatively correlated with their gene expression, whereas a positive correlation was observed for *TTC21A*.

**Figure 5:**
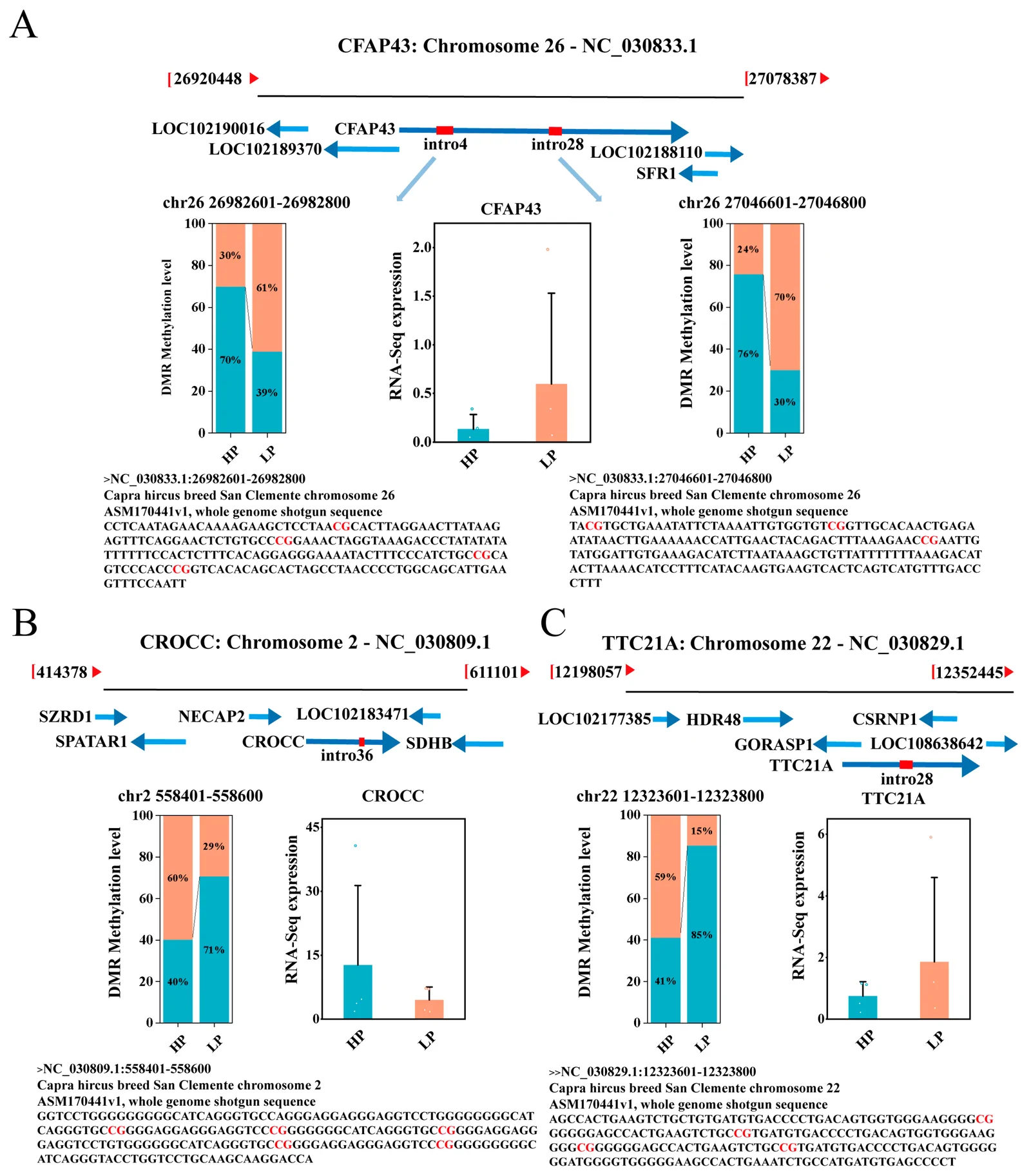
Methylation and expression profiles of key candidate genes. (**A**–**C**) Genomic organization of *CFAP43* (**A**), *CROCC* (**B**) and *TTC21A* (**C**). Red rectangles mark the positions of DMRs. Bar graphs show the methylation levels and relative gene expression in HP and LP groups. The nucleotide sequences of target DMRs are presented below, with CpG sites highlighted in red.

The distribution of methylation sites and regulatory features for each gene is depicted in Figure 5. *CFAP43* contained two DMRs: DMR1, located in intron 4, encompassed 4 CpG sites and exhibited a significantly higher methylation rate in the HP group (70%) compared to the LP group (39%). DMR2, located in intron 28, contained 3 CpG sites with a methylation rate of 76% in the HP group versus 30% in the LP group. Consistent with the hypermethylation state in its promoter-proximal regions, *CFAP43* expression was significantly downregulated in the HP group (Figure 5A). The DMR for *CROCC*, located in intron 36, contained 5 CpG sites. Its methylation rate was lower in the HP group (40%) than in the LP group (71%), and its expression level was correspondingly upregulated (Figure 5B). For *TTC21A,* the DMR in intron 28 contained 4 CpG sites. The methylation rate was 41% in the HP group and 85% in the LP group. *TTC21A* expression was significantly downregulated in the HP group, suggesting its expression may be positively regulated by methylation (Figure 5C).

In summary, the four identified DMRs (comprising 16 methylation sites) within the *CFAP43*, *TTC21A*, and *CROCC* genes exhibited significant methylation differences associated with reproductive traits. These sites hold theoretical value as potential epigenetic markers for the genetic improvement of Jining Grey Goats.

## 4. Discussion

Litter size in goats is a complex quantitative trait governed by the combined regulation of multiple genes, signaling pathways, and environmental factors [7]. In recent years, the role of epigenetic regulation, particularly DNA methylation, in trait formation has garnered increasing attention, yet its specific mechanisms in goat prolificacy remain unclear. This study presents the first genome-wide DNA methylation profile of uterine tissue from Jining Grey Goats exhibiting significant divergence in litter size. By integrating multi-omics data and experimental validation, we identified key epigenetic regulators influencing litter size.

First, we systematically constructed a genome-wide DNA methylation landscape of Jining Grey Goats uterine tissue. Our analysis identified 880 DMRs and 274 DMGs in the uterine tissues of Jining Grey Goats with different litter sizes. The methylation level of DMRs was significantly higher in the high-prolificacy (HP) group than in the low-prolificacy (LP) group. The genome-wide methylation pattern exhibited notable sequence context specificity, dominated by the CG context, where methylation levels were substantially higher than those in CHG and CHH contexts. Methylation distribution across different genomic functional elements displayed characteristic patterns: gene bodies were hypermethylated, while regions flanking promoters were hypomethylated. This pattern is highly consistent with previous reports in other goat tissues, including ovary [12,14,25], hypothalamus [25], and muscle [26]. Furthermore, similar distribution patterns have been observed in other mammals, such as the ovaries of Hu sheep [27] and Landrace pigs [28], the spleen and liver of Yunnan humped cattle and Holstein cattle [29], and tissues of the hypothalamic-pituitary-ovarian axis in pigs [30]. This conservation across tissues and species underscores the high evolutionary stability of DNA methylation regulatory mechanisms. Notably, this study is the first to characterize this pattern in the goat uterus, a key reproductive organ, providing fundamental data for epigenetic research on reproductive traits in ruminants.

A pivotal finding of this study is the identification of 21 DEG-DMGs through integrated multi-omics analysis. Combined with protein–protein interaction (PPI) network analysis, *CFAP43*, *TTC21A*, and *CROCC* emerged as key regulatory candidates. *CFAP43* (also termed WDR96), located on goat chromosome 26, belongs to the WD repeat protein family, known for participating in protein interactions and complex assembly. Previous studies indicate that *CFAP43* is predominantly expressed in human and mouse testis, where it maintains flagellar axoneme stability (particularly central microtubules and radial spokes) and regulates normal sperm and trypanosome flagellar motility. Biallelic pathogenic mutations in *CFAP43* cause multiple morphological abnormalities of the flagella (MMAF) and male infertility in humans [31]. As essential organelles, cilia and flagella are widely involved in cell motility, signal transduction, and material transport. The *CFAP43* protein plays a critical role in the structure and function of cilia/flagella, regulating ciliary beating patterns and efficiency, and influencing reproductive, respiratory, and neurological functions. It has been proposed as an important candidate gene for “ciliopathy-related multisystem disorders” [32]. Functional studies in mice and Xenopus laevis further demonstrate that *CFAP43* deficiency leads to abnormal ciliary motility, thereby impairing normal physiological functions [32,33]. Research in Shanbei White Cashmere goats also confirms that its mutation is significantly associated with litter size [34], indicating the potential of *CFAP43* for marker-assisted selection in goat breeding. Importantly, our previous work in Jining Grey Goat ovarian tissue also identified *CFAP43* as a differentially expressed and methylated gene, with the DMR at chr26:27046601–27046800 similarly detected in the ovary [14]. Moreover, functional defects in other CFAP family members (e.g., *CFAP410*) are associated with ciliary structural abnormalities, such as retinopathy and skeletal dysplasia [35,36], further highlighting this family’s general importance in ciliary function maintenance and suggesting that *CFAP43* is under conserved epigenetic regulation in female goat reproduction.

The *TTC21A* gene, also known as *IFT139A*, encodes a protein containing multiple tetratricopeptide repeat (TPR) motifs. As a component of the IFT-A complex, it is essential for ciliary structure stability and function [37]. Zhu et al. [38] found that *CEP78* participates in regulating ciliary axoneme and centriole elongation by directly interacting with *IFT20* and *TTC21A*. Although TTC21A was initially characterized in testicular and spermatogenic cells, where homozygous mutations cause multiple morphological abnormalities of the flagella (MMAF) and male infertility [39], it is important to emphasize that *TTC21A* is a ubiquitously expressed component of the IFT-A complex in all ciliated cells, not restricted to sperm. In the female reproductive tract, multiciliated epithelial cells are abundant in the oviduct and uterine endometrium, where ciliary beating is essential for oocyte pickup, embryo transport, and endometrial receptivity [40,41]. Although sperm flagella and uterine motile cilia differ in their motility patterns and functional contexts, both rely on the conserved IFT machinery—including the IFT-A complex containing *TTC21A*—to build and maintain their axonemal structure. The present study is the first to identify a DMR in its 28th intron in uterine tissue. The protein–protein interaction between *TTC21A* and *CFAP43* was predicted by the STRING database based on experimental evidence from co-immunoprecipitation and affinity purification-mass spectrometry studies in ciliated cells [31,39]. At the mechanistic level, *TTC21A* (also known as *IFT139A*) is a core subunit of the intraflagellar transport (IFT)-A complex, which mediates retrograde trafficking from the ciliary tip to the cell body, whereas *CFAP43* (also known as *WDR96*) is an axonemal central-pair/radial spoke protein essential for ciliary and flagellar ultrastructure. During ciliary assembly and maintenance, the IFT-A cargo complex physically engages with axonemal structural proteins including *CFAP43* to facilitate their transport and turnover along the ciliary axoneme, providing a mechanistic basis for their functional cooperation. However, it should be noted that the direct physical interaction between *TTC21A* and *CFAP43* in goat uterine tissue was inferred from the STRING prediction rather than experimentally validated in the present study; future co-immunoprecipitation or proximity ligation assays in uterine epithelial cells would confirm this interaction. These findings suggest that beyond its established role in male gametogenesis, *TTC21A* may also participate in female reproductive tract ciliary function and thereby contribute to uterine receptivity and litter size.

The *CROCC* gene encodes Rootletin, the core structural protein of the ciliary rootlet, which forms the molecular basis for centriole cohesion and ciliogenesis [42,43,44]. This study identified *CROCC* as a key gene associated with fertility in Jining Grey Goat uterine tissue, with its expression negatively regulated by DNA methylation. Previous research indicates that *CROCC* is crucial for intercentriolar fiber function and ciliary assembly [42]. Rootletin deficiency leads to loss of the ciliary rootlet, aberrant centriole separation, and ciliary dysfunction [43]. Furthermore, as a key downstream gene of Mfn2 in ciliary regulation, *CROCC* downregulation reduces Rootletin protein levels, thereby affecting ciliary structure [44]. Mutations in *CROCC* also cause ciliary abnormalities and disrupt Hedgehog signaling [45]. Therefore, the high expression of *CROCC* in the HP group may stabilize the ciliary rootlet, ensuring normal uterine cell signaling critical for embryo implantation. Interestingly, *CFAP43*, *TTC21A*, and *CROCC* all converge on ciliary structure and function. Cilia are ubiquitous in the female reproductive tract epithelium, and their structural integrity and normal function are vital for gamete transport, embryo movement, and signal transduction [46]. In the uterus, primary cilia act as signaling hubs, precisely regulating endometrial decidualization and embryo implantation by mediating pathways such as Hedgehog and GPCR signaling [40,41]. Defects in the ultrastructure of multiciliated endometrial glands can compromise endometrial receptivity, leading to reproductive disorders [47]. Hence, we propose a central hypothesis: DNA methylation regulates the expression of cilia-related genes in the uterus, thereby influencing the prolificacy of Jining Grey Goats.

The sample size of this study was relatively small, which may affect statistical power; therefore, future studies with larger sample sizes are needed to validate these findings. In addition, functional validation via gene knockdown or overexpression experiments in endometrial cells is crucial to confirm the regulatory roles of these candidate genes and their methylation sites in uterine biology.

It should be acknowledged that litter size is a complex quantitative trait influenced by multiple biological processes, including ovulation rate, fertilization success, embryo survival, implantation efficiency, and endometrial receptivity. The present study focused exclusively on DNA methylation differences in uterine tissue, and therefore our findings may not fully capture the contributions of ovarian function, oviductal transport, or systemic hormonal regulation to litter size. In addition, the functional relevance of the identified DMRs and candidate genes in uterine cilia remains to be validated through in vitro and in vivo experiments, such as overexpression/knockdown of these genes in endometrial epithelial cells and assessment of ciliary beating and embryo implantation. Future studies integrating ovarian, oviductal, and uterine methylomes, combined with functional validation, will be necessary to fully elucidate the epigenetic architecture underlying goat prolificacy.

In summary, this study constructed a genome-wide DNA methylation profile of Jining Grey Goat uterine tissue and, through integrated multi-omics analysis, identified three key candidate genes (*CFAP43*, *TTC21A*, and *CROCC*) associated with reproductive traits. We found that their expression in uterine tissue is regulated by DNA methylation and that they are all involved in maintaining ciliary structure and function. Based on these findings, we propose a potential mechanism: DNA methylation may affect uterine ciliary function and endometrial receptivity by regulating the expression of key genes, thereby contributing to litter size differences in Jining Grey Goats. The 16 methylation sites identified within these genes may serve as key molecular markers. Our results provide novel theoretical insights and research directions for understanding the mechanisms underlying goat prolificacy and offer potential candidate targets for future functional studies and molecular breeding research.

## 5. Conclusions

This study presents the first genome-wide DNA methylation profile of uterine tissue in Jining Grey Goats through WGBS. Integrated with transcriptome data, we identified three differentially methylated genes—*CFAP43*, *TTC21A*, and *CROCC*—along with 16 specific methylation sites that are closely associated with prolificacy traits in this breed. Our findings provide novel insights into the epigenetic regulation of litter size in goats and highlight candidate genes for future functional validation and potential application in marker-assisted breeding strategies.

## Figures and Tables

**Table 1:** Primers sequences of DNA methylation-related genes.

Region	Sequences (5′-3′)	Product Length (bp)	Associate Gene	Gene Bank
chr8:90694948-90695222	F:TTGTAGTTAAGGGAGTAAGGGTAAAGAG	295	*PLPPR1*	NC_030815.1
	R:AACAAAAAACCCCTATACACACAAA			
chr8:90700916-90701147	F:TGTTAGAAATTAGGTTTTGAATGTTAGAA	232	*PLPPR1*	NC_030815.1
	R:CTAACCAAAAAAATCACTATAAACC			
chr26:26982573-26982794	F:GGAGTTTTTTAGGAGTTTATTTAGAAT	222	*CFAP43*	NC_030833.1
	R:AACTTCAATACTACCAAAAATTAAACTAAT			
chr17:35331809-35332086	F:AGGTGGAAGAAAATTATTTTTAAAGTAAAG	278	*TRPC3*	NC_030824.1
	R:ATCCTCCCCAACAATTCTACTAATT			

## Data Availability

All sequencing data are available in the NCBI SRA under BioProject PRJNA1357378 (WGBS data) and PRJNA1347776 (RNA-seq data).

## Supplementary Materials

The following supporting information can be downloaded at https://www.mdpi.com/article/10.3390/ani16182935/s1. Table S1. Whole genome DNA bisulfite sequencing data; Table S2. Co-expressed genes by combined analysis of WGBS and RNA-Seq in uterine tissues of Jining Grey Goats; Table S3. WGBS and RNA-seq results of three genes in uterine tissues of Jining Gray Goats; Table S4. The enrichment status of key DMG-DEG in the uterine tissues of Jining Grey Goats across different prolificacy groups; Table S5. 21 DMG-DEGs identified by integrated WGBS and RNA-seq analysis; Figure S1. Pearson Correlation of WGBS Methylation Profiles.

## Abbreviations

The following abbreviations are used in this manuscript:

NCBI	National Center for Biotechnology Information
HP	High-prolificacy
LP	Low-prolificacy
HP_Us	High-prolificacy uterus
LP_Us	Low-prolificacy uterus
DEGs	Differentially expressed genes
DMGs	Differentially methylated genes
WGBS	Whole Genome Bisulfite Sequencing
BSP	Bisulfite Sequencing PCR (BSP)
