# Supplementary material for: Genome-Wide Analysis of DNA Methylation Profiles on Jining Grey Goat Uterus Associated with Prolificacy Using Whole-Genome Bisulfite Sequencing
Source: Animals (Basel). 2026 Sep 18;16(18):2935. doi: 10.3390/ani16182935 (PMC13603397; doi:10.3390/ani16182935)
Supplement: Supplementary file 1 [file animals-16-02935-s001.zip › animals-4484557-supplementary.pdf]

Supplementary Table S1. Whole genome DNA bisulfite sequencing data

Groups	sample	Clean Reads	Q30 (%)	Total Mapping Reads（%）	Unique Mapping Ratio (%)	Bisulfite Conversion Rate(%)	Total_mC (%)
HP	HP_U1	1312984878	89.5	86.66	80.87	99.6	2.9
	HP_U2	1363412686	93.34	86.68	80.27	99.56	2.83
	HP_U3	1314758434	90.31	89.73	82.84	99.6	3.07
	HP_U4	1009458200	87.97	89.78	83.13	99.56	4.08
LP	LP_U1	1162418192	91.97	84.92	79.2	99.52	3.34
	LP_U2	1347278924	93.14	85.38	79.57	99.58	2.9
	LP_U3	1072478832	90.99	80.21	74.38	99.52	3.58
	LP_U4	1058837846	88.69	77.6	72	99.54	3.63

Clean Base (Gb):The number of Clean Base throughout the sequence Clean Reads The number of clean reads

Unique Mapping Ratio (%):The number of clean reads matched to the reference genome relative to the total clean reads

Bisulfite Conversion Rate (%):The number of clean reads matched to the reference genome relative to the amount of methylation of the clean reads matched to the reference genome

Total mC (%) The number of clean reads matched to the reference genome relative to the amount of methylated cytosine within the clean reads matched to the reference genome

Supplementary Table S2. Co-expressed genes by combined analysis of WGBS and RNA-Seq in uterine tissues of Jining Grey Goats

Group	Genes	Gene counts
Hyper DMG-Hyper DEG	*LOC102185549*、*LOC102181111*、*SUSD4*、*FSTL5*、*JAKMIP3*、*LOC102174011*、*LOC108634920*	7
Hyper DMG-Hypo DEG	*OTOG*、*TRPC3*、*CFAP43*、*CSMD2*、*GRIP1*、*LOC102168532*、*PLPPR1*、*LOC108635527*、*ZNF804B*	9
Hypo DMG-Hyper DEG	*CROCC*	1
Hypo DMG-Hypo DEG	*ZNF804B*、*LOC108637248*、*TTC21A*、*CD5L*、*MUC19*	5

Supplementary Table S3. WGBS and RNA-seq results of three genes in uterine tissues of Jining gray goats

Gene	DMR	Region	Meth-Direction	Meth Diff	Q-Value	mRNA Log2 Fold Change	mRNA Qvalue
CFAP43	chr26_26982601_26982800	intron	Hyper	0.31	3.55E-04	-2.102349628	3.75E-27
TRPC3	chr17_35332001_35332200	intron	Hyper	0.46	1.54E-03	-1.173584533	4.37E-11
PLPPR1	chr8_90695001_90695200	UP3K	Hyper	0.31	1.00E-07	-1.336335501	7.95E-05
	chr8_90700801_90701000	intron	Hyper	0.49	1.70E-11		

Supplementary Table S4. The enrichment status of key DMG-DEG in the uterine tissues of Jining Grey goats across different prolificacy groups

Gene	Functional Enrichment			P -value	DMR	DMR context	DMR element	Differential methylation levels	Meth-Direction	Log2FC	Regulated
CFAP43	MF	GO:0005515	protein binding	6.9675E-05	chr26 26982601_26982800	CG	intron;repeat;gene	0.309	Hyper	-2.102349628	down
					chr26 27046601_27046800	CG	intron;repeat;gene	0.4583	Hyper		
TTC21A	MF	GO:0005515	protein binding	6.9675E-05	chr22 12323601_12323800	CG	intron;gene	-0.4407	Hypo	-1.288433262	down
CROCC	CC	GO:0005886	plasma membrane	0.03142421	chr2 558401_558600	CG	intron;gene	-0.3045	Hypo	1.545548235	UP
