# Supplementary material for: Genome-Wide Analysis of DNA Methylation Profiles on Jining Grey Goat Uterus Associated with Prolificacy Using Whole-Genome Bisulfite Sequencing
Source: Animals (Basel). 2026 Sep 18;16(18):2935. doi: 10.3390/ani16182935 (PMC13603397; doi:10.3390/ani16182935)

Pearson Correlation of WGBS Methylation Profiles
(Chromosome−level CG methylation rate)

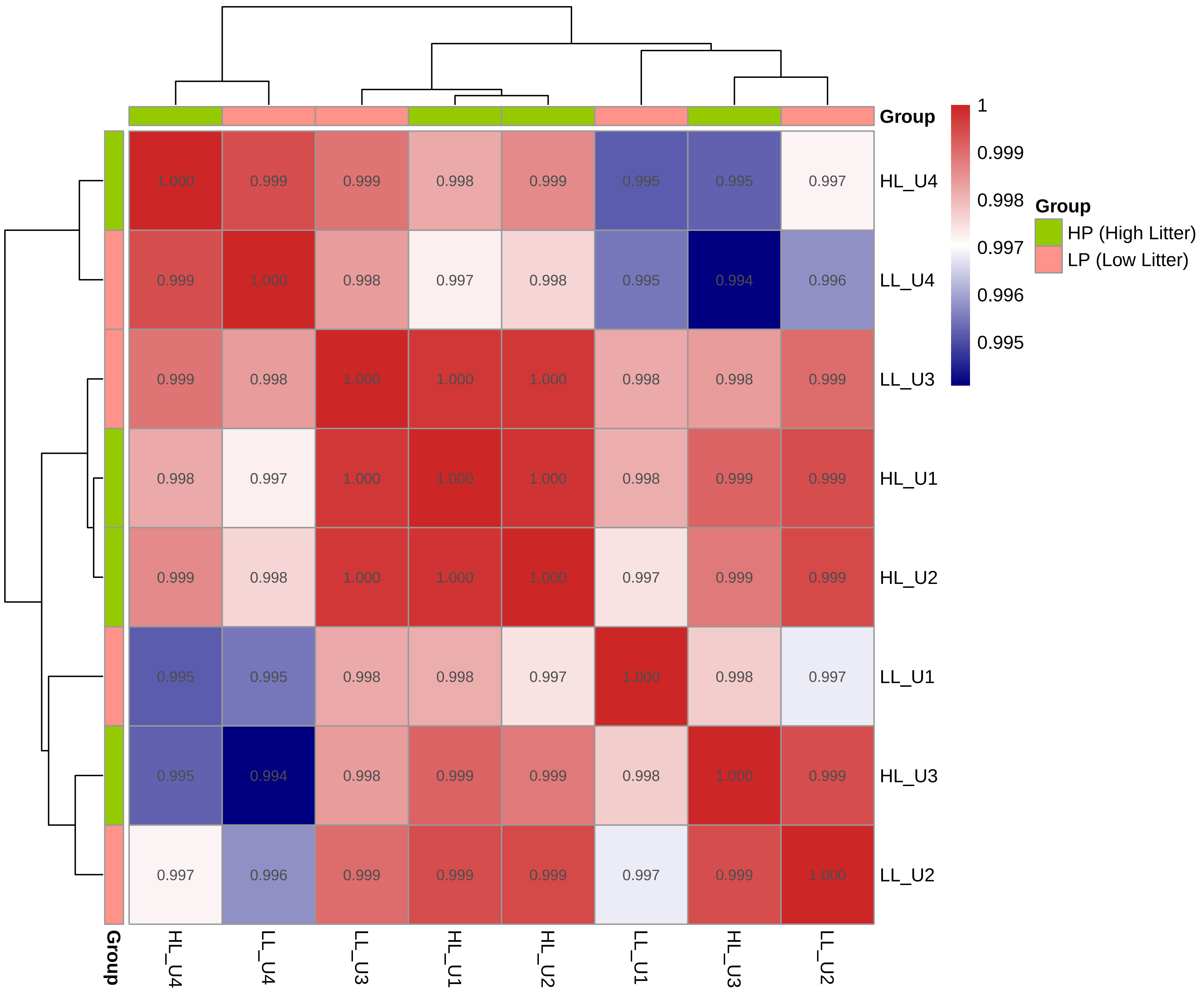

Supplement: Supplementary file 1 [file animals-16-02935-s001.zip › Figure S1.pdf]
